# Did Equity of Reproductive and Maternal Health Service Coverage Increase during the MDG Era? An Analysis of Trends and Determinants across 74 Low- and Middle-Income Countries

**DOI:** 10.1371/journal.pone.0134905

**Published:** 2015-09-02

**Authors:** Sarah Alkenbrack, Michael Chaitkin, Wu Zeng, Taryn Couture, Suneeta Sharma

**Affiliations:** Health Policy Project, Center for Policy and Advocacy, Futures Group, Washington, DC, United States of America; London School of Economics, UNITED KINGDOM

## Abstract

**Introduction:**

Despite widespread gains toward the 5^th^ Millennium Development Goal (MDG), pro-rich inequalities in reproductive health (RH) and maternal health (MH) are pervasive throughout the world. As countries enter the post-MDG era and strive toward UHC, it will be important to monitor the extent to which countries are achieving equity of RH and MH service coverage. This study explores how equity of service coverage differs across countries, and explores what policy factors are associated with a country’s progress, or lack thereof, toward more equitable RH and MH service coverage.

**Methods:**

We used RH and MH service coverage data from Demographic and Health Surveys (DHS) for 74 countries to examine trends in equity between countries and over time from 1990 to 2014. We examined trends in both relative and absolute equity, and measured relative equity using a concentration index of coverage data grouped by wealth quintile. Through multivariate analysis we examined the relative importance of policy factors, such as political commitment to health, governance, and the level of prepayment, in determining countries’ progress toward greater equity in RH and MH service coverage.

**Results:**

Relative equity for the coverage of RH and MH services has continually increased across all countries over the past quarter century; however, inequities in coverage persist, in some countries more than others. Multivariate analysis shows that higher education and greater political commitment (measured as the share of government spending allocated to health) were significantly associated with higher equity of service coverage. Neither country income, i.e., GDP per capita, nor better governance were significantly associated with equity.

**Conclusion:**

Equity in RH and MH service coverage has improved but varies considerably across countries and over time. Even among the subset of countries that are close to achieving the MDGs, progress made on equity varies considerably across countries. Enduring disparities in access and outcomes underpin mounting support for targeted reforms within the broader context of universal health coverage (UHC).

## Introduction

As the Millennium Development Goals (MDGs) era draws to a close, there is evidence that the least progress has been made toward the maternal health goal (MDG 5) which seeks to reduce the maternal mortality ratio (MMR) by three quarters between 1990 and 2015 to achieve universal access to reproductive health by 2015 [1]. Despite widespread gains in access to antenatal care and skilled delivery attendance, contributing to a 45% global reduction in MMR over the past quarter century, nearly 300,000 women died from pregnancy and childbirth-related causes in 2013 [2]. In 17 countries, MMR has actually increased—in some cases substantially [2]. Moreover, pro-rich inequalities in maternal health (MH) are pervasive [3–6], and are much greater than inequalities for immunization coverage or treatment of childhood illnesses [7].

A major criticism of the MDGs is that a preoccupation with aggregate outcomes has detracted from much needed attention to the poor and vulnerable, groups that are more difficult to reach than the rest of the population [8]. Enduring disparities in access and outcomes underpin mounting support for universal health coverage (UHC), which will likely serve as the umbrella health goal for the post-2015 development agenda. Similarly, *The Lancet Commission on Investing in Health* expects progress toward UHC to propel its vision for a “grand convergence” in health, in which by 2035 the infant, child, and maternal mortality rates in low- and middle-income countries would fall to levels commensurate with today’s best performing middle-income countries, thereby preventing about 10 million premature deaths [9]. Although UHC is a prerequisite for an equitable health system, UHC strategies will not automatically translate to improved access for the poor [8]. In fact, the evidence suggests that the poor will be the last to be covered when an untargeted health intervention is rolled out, leading to greater pro-rich inequalities [10]. This notion that focusing on global targets exacerbates inequalities gave rise to the concept of “progressive universalism,” an approach to health systems strengthening in which the poor gain at least as much in terms of health outcomes and financial risk protection as everyone else through every step toward UHC [8]. Jamison et al. (2013) offer two variants, one in which publicly financed benefits packages would be designed to address health issues disproportionately burdening the poor, and another in which benefits would be broader but some degree of cost-sharing would be imposed on the non-poor [9]. Both approaches resonate with Marmot et al. (2010)’s proposal of “proportionate universalism” to address health inequalities in England [11], a principle embraced by Thomsen et al. (2011) for application in developing countries as well [12]. Similarly, it will be important to design targeted policies that address the structural drivers of inequity and ensure equal opportunity [6].

As countries move toward UHC it will be important to better understand how they are making progress not only in their overall coverage, but also in terms of equity. Moreover, it will be beneficial to understand *why* maternal health services are more equitable in some countries than in others and what policy factors are associated with higher equity. The number of country-specific studies highlighting the scale of poor-rich inequalities in reproductive and maternal health in developing countries is enormous and growing, and much of it is summarized in a handful of systematic reviews [3, 7, 13]. Other reviews summarize the impact of targeted interventions—most of which are demand-side financing schemes—aimed to improve equity of MH [5, 6]. However, none of these multi-country reviews examines the *determinants* of equity of coverage at a macro level. Given the global consensus that political commitment is an ingredient for successful health sector reforms [9], it is also important to understand what policy factors explain a country’s progress, or lack thereof, toward more equitable RH and MH coverage. There is value in examining the relative importance of specific policy factors, like the share of government budgets allocated to health and the extent of prepayment in the health system, in achieving equity.

This study examines the equity of RH and MH coverage between countries and over time, looking at the decade preceding the Millennium Declaration and the subsequent 14-year MDGs era. We use descriptive analysis to present trends across four indicators of RH and MH coverage–contraceptive prevalence rate (modern methods), demand met for contraception (modern methods), antenatal care, and facility delivery. We then conduct multivariate analysis to examine the relative importance of policy factors, such as political commitment, governance, and level in prepayment, in determining countries’ progress toward greater MH equity. Our analysis is based on panel data compiled from Demographic and Health Surveys (DHS) and other secondary sources. We interpret the results by drawing on the health policy and financing literature to discuss how specific reforms such as introduction of UHC, community-based health insurance (CBHI), health equity funds, and removal of user fees, contributed to improvements in equity.

## Methods

### Ethics

We used secondary data analysis of DHS surveys and therefore ethics approval was not required. The data contained no household identifiers and the secondary data are publicly available.

### Data sources and indicators

To examine trends in equity of RH and MH service coverage we compiled a multi-country dataset using the DHS Program’s STATcompiler tool [14], which provides consolidated data from 223 DHS, reproductive health surveys, and malaria indicators surveys conducted in 87 countries between 1986 and 2013. The data measure coverage rather than just utilization because the RH and MH indicators account for ‘utilization relative to need’ given that the denominator includes women with a need for the respective services. DHS include data on socioeconomic status (SES), measured with a household asset index constructed using principal components analysis [15]. Despite its limitations [16, 17], the asset index has been used widely as a measure of socioeconomic position in low- and middle-income countries. In our dataset, we included coverage data for four MH services indicators, disaggregated by wealth quintiles as provided by STATcompiler. DHS’s wealth quintiles cover countries’ entire populations, not just the sub-populations of those needing RH and MH services. DHS did not begin collecting household wealth data until 1990, so our analysis of equity relies on 190 surveys from 74 countries between 1990 and 2013 (S1 Table). For the reason of consistency, we did not include Multiple Indicator Cluster Survey (MICS) in this study.

We examined indicators covering use of modern family planning methods, antenatal care, and facility delivery, as these are among the key RH and MH services that countries endeavor to improve in pursuit of MDG 5. Additionally, all women need these services to maintain and enhance their (and their children’s) health and welfare. We used two family planning indicators: the prevalence rate of modern contraceptive methods and the share of demand met for modern contraceptives. Modern methods include pills, intrauterine devices (IUDs), injections, male and female condoms, implants, lactational amenorrhea method (LAM), male and female sterilization, diaphragms, and foams and jellies. Both were measured among women aged 15 to 49 currently married or in union. Demand met is calculated from the prevalence of unmet need for family planning, which includes unmet need for limiting (women whose current or latest pregnancy was unwanted or who want no more children) and for spacing (women who do not want their next child for at least two years or who are undecided or unsure about the timing or desirability of future pregnancy) [18]. Demand met is the number of women using contraception divided by the sum of the number of current users and the number of women with unmet need. We also examined two antenatal and delivery service indicators: the share of pregnant women with any antenatal care from trained health professionals, including physicians, nurses, midwives, and health workers, and the share of births that occurred in health facilities. For each variable we used data on all reported births in the three years preceding each survey. Aside from the CPR variable, the indicators account for level of need within each wealth quintile, which is important for assessing vertical equity—the principle that individuals who are unequal should be treated differently according to their level of need [19].

### Descriptive analysis

The first portion of our analysis focused on trends in equity of MH service coverage across countries and over time. Due to space limitations, we focused more on relative equity, acknowledging that it is also important to consider how the absolute level of equity is changing over time. To measure relative equity we used the concentration index, which is a standard measure of equity. We applied the formula provided by O’Donnell et al. [19] for each MH service using coverage data grouped by wealth quintile. The value of the concentration index (CI) ranges from -1 to 1, with 0 representing equality, and positive values indicating a pro-rich skew in the indicator. We also compared equity of facility delivery to the maternal mortality ratio (MMR) with data from the World Health Organization (WHO)’s Global Health Observatory Data Repository [20].

### Multivariate analysis

In the second portion of our analysis, we explored the determinants of equity of MH service coverage. Typically scholars attempting to explain determinants of inequality conduct decomposition analysis, as described by O’Donnell et al. [19], to identify the drivers of unequal health status or service coverage. Such analysis requires disaggregated data, whether across wealth, education, or ethnic dimensions, not only for the outcome of interest, such as maternal mortality, but also for its correlates. This approach is well suited to those correlates of equity for which data are available at the individual or household level. However, it is also of interest to examine the relationship between equity and less divisible factors, like a country’s policy environment. For instance, it would be difficult to conceptualize (much less quantify) political commitment or governance at the micro level. Thus, rather than decomposing our concentration indices, we used them as dependent variables in a multivariate regression model.

Health inequities within and between countries stem from various social determinants of health [21]. With country-year as our unit of observation, we focused on the macro-level social determinants, features of what the WHO’s *Commission on Social Determinants of Health* described as a country’s “socioeconomic-political context” [22]. Contextual factors include macroeconomic policy, governance, social and sectoral policies, and social norms. In particular we collected data on (1) country socioeconomic status (income and education); (2) health policy indicators (political commitment and level of prepayment); and (3) governance. Our random-effects model controls for time-invariant factors.

To measure country socioeconomic statistics we collected data on gross domestic product (GDP) per capita and secondary school enrollment from the World Bank’s World Development Indicators [23]. We considered two features of health sector policy. First, we used the share of governments’ total expenditure that was allocated to health as a proxy for political commitment to the health sector [24]. Second, we characterized the health financing structure by first dividing total health spending into out-of-pocket and prepaid shares. One of the key recommendations for improving health equity is increasing the prepaid share of total health expenditure. We also divided prepaid expenditures into private and public spending. We hypothesized that, compared with OOP, a higher share of public or private prepaid expenditure in a country would be associated with greater equity. We obtained all health expenditure data from the WHO’s Global Health Expenditure Database, which draws on countries’ self-reported National Health Accounts [25]. Finally, we sought a broad governance indicator that would measure the state’s role in redistribution of health resources to promote equity, including as guarantor of rights and services, facilitator of policy to improve health, and custodian of key health and population data [26]. Other key governance features include empowerment and voice and lack of corruption [21, 27]. Other studies suggest that poor governance hinders the effectiveness of public health spending [28–30], and may explain the lack of association between public health expenditures and health outcomes [31]. We therefore collected data on voice and accountability, rule of law, government effectiveness, control of corruption, regulatory quality, and political stability and absence of violence from the World Bank’s Worldwide Governance Indicators (WGI) database [32]. Initially WGI only published data in even years, so for the first several odd years (1997, 1999, and 2001) we interpolated values by taking the arithmetic mean of the surrounding two years. The six WGI indices are highly correlated, so we conducted factor analysis and abstracted one factor score with an Eigenvalue greater than 1 to be included in the multivariate analysis. This factor score was driven most by measures of government effectiveness and rule of law, and this factor explained more than 90% of the variation across all six indicators.

We constructed a panel dataset by combining these data with our computed concentration indices. We then fit a series of generalized least squares models with random effects. We confirmed the appropriate use of random effects using the Hausman test. The null hypothesis, which we failed to reject (p = 0.37), was that random effects were a better fit to our data than fixed effects. All statistical analyses were run in Stata 13 (S1 Data). All statistical analyses were run in Stata 13. Definitions and data sources for the variables included in our regressions can be found in S3 Table.

## Results and Discussion

### Descriptive analysis

**Fig 1:**
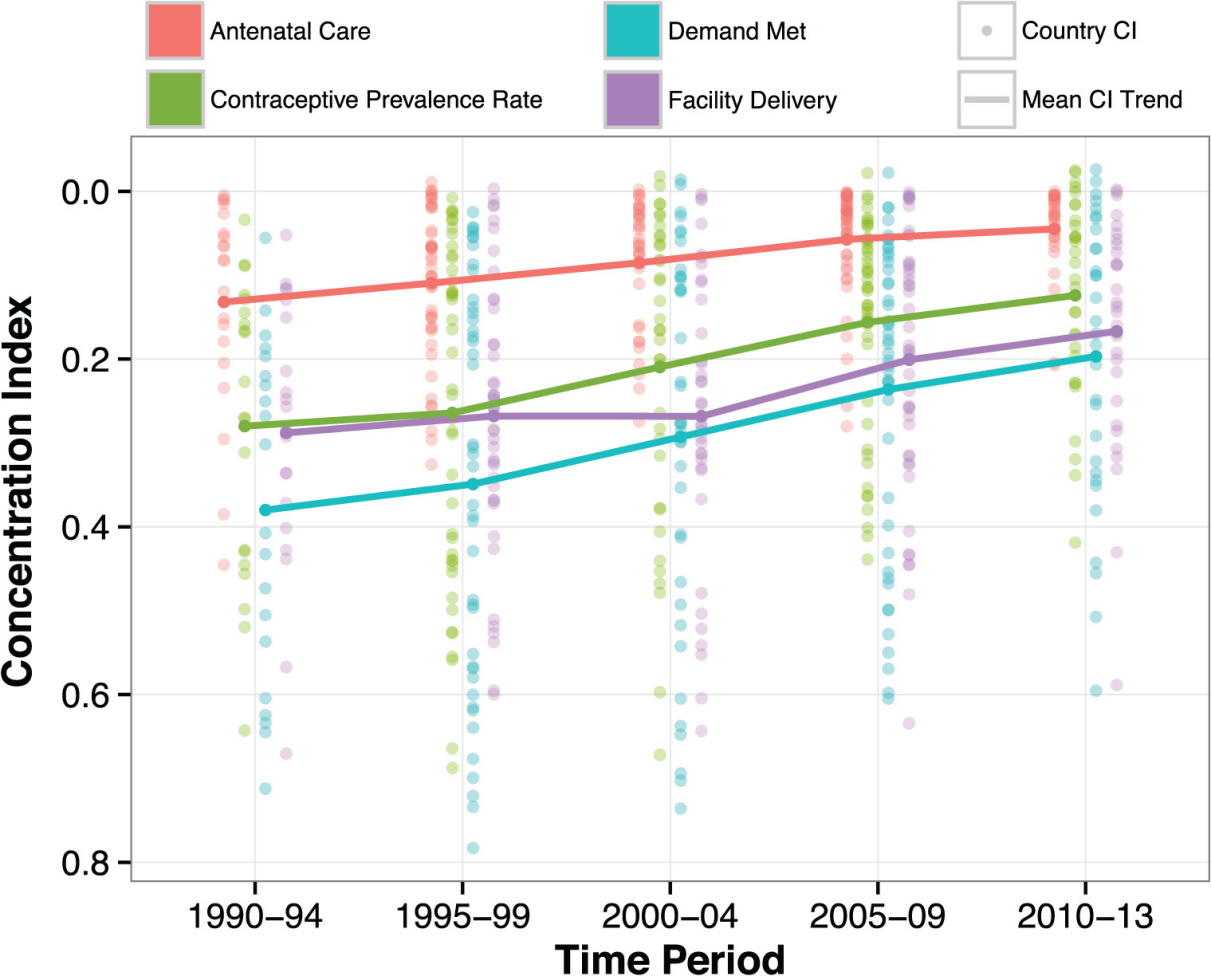
Equity of reproductive and maternal health service coverage, full sample of countries, 1990–2013.

Fig 1 shows the global trends in equity of service coverage for four RH and MH indicators. We computed the average concentration index for each indicator across five time periods, prompting a handful of basic observations. First, relative equity of coverage for all four services has improved substantially and continually, except for a stalling of progress in the early 2000s for facility delivery. This time period coincides with the implementation of user fees in many countries, particularly in Africa, and may be a reflection of the reduced utilization of health services that was documented in multiple countries following that change [33–35]. Given the large number of African countries in our sample, this trend may be influencing the overall trends in the sample. While Fig 1 does not show changes in absolute equity, absolute equity has also improved during this time period.

A second observation of Fig 1 is that despite progress made in the last 25 years, inequities in coverage persist across all four RH and MH services. Third, just like in 1990, demand met for modern contraceptives is the least equitable of the four services. ANC, in contrast, is the most equitable. In fact, in the latest time period, use of ANC was approaching perfect equity, although we are cautious not to place too much weight on this finding given that the indicator only measures use of at least one visit. While closing the socioeconomic gap in access to at least one ANC visit is encouraging, the quality of ANC still varies dramatically across and within countries, and the standard of care is four ANC visits per pregnancy [36].

**Fig 2:**
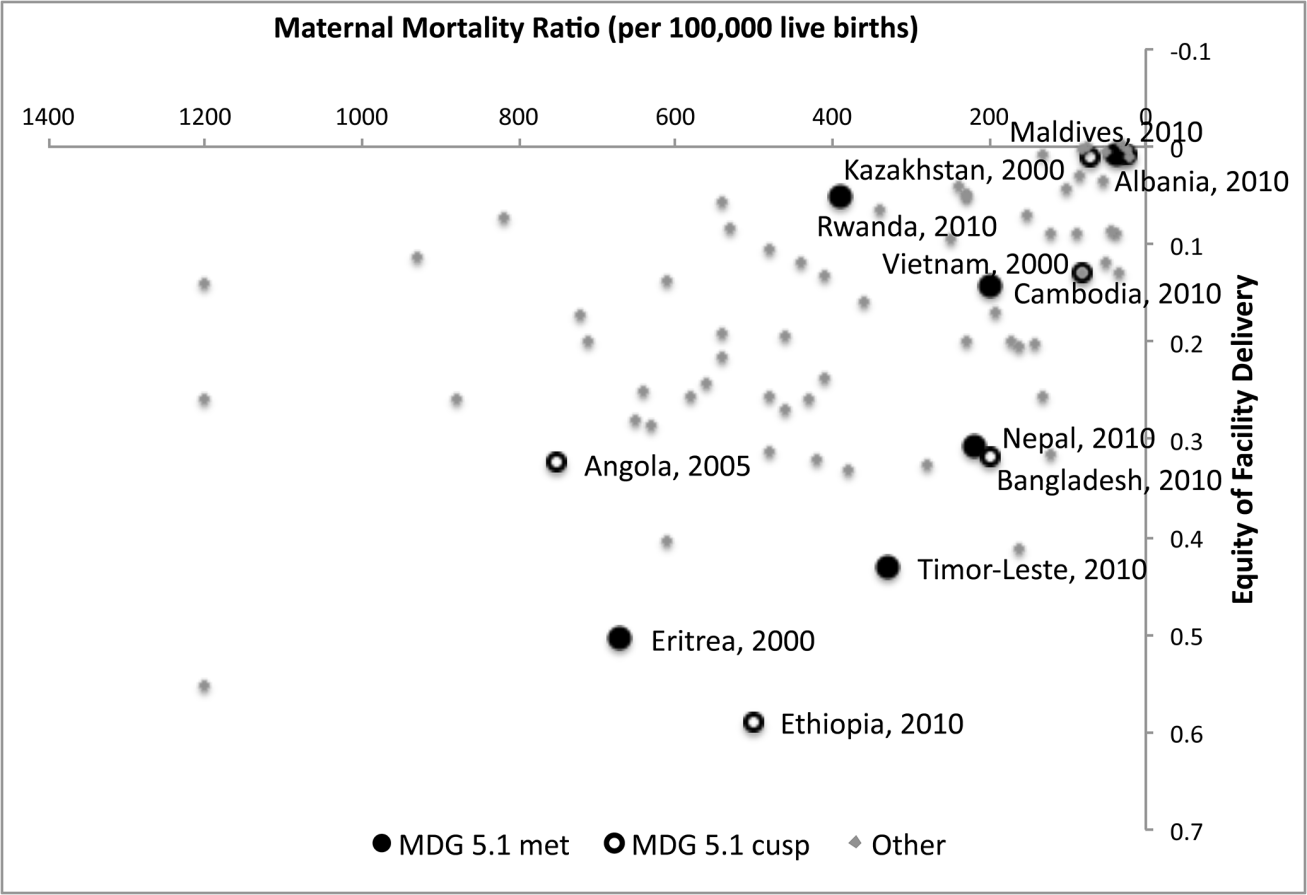
Maternal mortality ratio vs. equity of facility delivery.

We also explored the relationship between equity of facility delivery and maternal mortality rate (MMR) (Fig 2). Equity of facility delivery was measured for each country in the most recent available DHS. The WHO reports country-level MMR for every five years from 1990 to 2010 and for 2013 [20]. A country’s equity of institutional delivery was matched with the MMR figure for the year closest to the survey, at most two years before or after. By 2013, six of the countries in our sample had achieved MDG target 5.1, the reduction of MMR by at least 75% from 1990 levels. These countries include: Maldives (93%), Cambodia (86%), Eritrea (78%), Timor-Leste (78%), Rwanda (77%), and Nepal (76%). We classified an additional six countries as those that were on the cusp of reaching the target in their latest DHS survey, having reduced MMR by at least 65% but falling short of the MDG target. These countries are: Kazakhstan (71%), Ethiopia (70%), Bangladesh (69%), Angola (67%), India (66%), and Moldova (66%). 10 additional countries had met the absolute target of 60 maternal deaths per 100,000 live births for countries with intermediate mortality level. These targets were laid out in the International Conference on Population and Development’s Programme of Action [37], although they were not included among the MDGs in the Millennium Declaration.

We observed a moderate correlation (r = 0.47) between a country’s equity of facility delivery and MMR (Fig 2). The results show that although these countries have all made great strides to reduce their MMR, they vary considerably in the extent to which they have tackled inequalities. Ethiopia, for example, achieved a 65% reduction in MMR between 2000 and 2011, even though socioeconomic inequity was still high in 2010 (concentration index for facility delivery in 2010 was 0.59). In fact, in this time period Ethiopia was among the least equitable countries across all four indicators. Bangladesh and Cambodia both had national MMRs of 200 in the last time period but different levels of equity of facility delivery, with Cambodia having much greater equity than Bangladesh. (Concentration indexes for facility delivery in Bangladesh and Cambodia were 0.317 and 0.144, respectively). Cambodia’s progress in improving equity likely had much to do with the country’s successful implementation of health equity funds, which target the poor and now reach a large portion of the poorest quintile [38]. Albania and Kazakhstan had among the lowest MMRs and the highest equity. While our study did not track neonatal mortality rates over time, the results are consistent with a recent 24-country study by McKinnon et al. (2014), which found that both neonatal mortality rates and socioeconomic inequalities in neonatal mortality fell over two decades [39].

### Equity trends

Given that the frequency and timing of DHS surveys varies across countries, we present general trends over time by region, using the World Bank’s geographic regional groupings. However, it is important to note that the sample includes only countries where at least one DHS survey has been conducted between 1990 and 2013, and therefore the results are not representative of the entire region. It is likely that the best performing regions will not have DHS surveys, as DHS surveys are typically implemented in areas that receive USAID support for programs in health and other relevant sectors. Similarly, there are some countries that may have low levels of equity but do not receive USAID support, and those countries would not be represented here. Thus, it is not possible to rank countries in terms of least or most equitable, but the findings are nonetheless useful for understanding trends in select countries.

#### Europe and Central Asia

**Fig 3:**
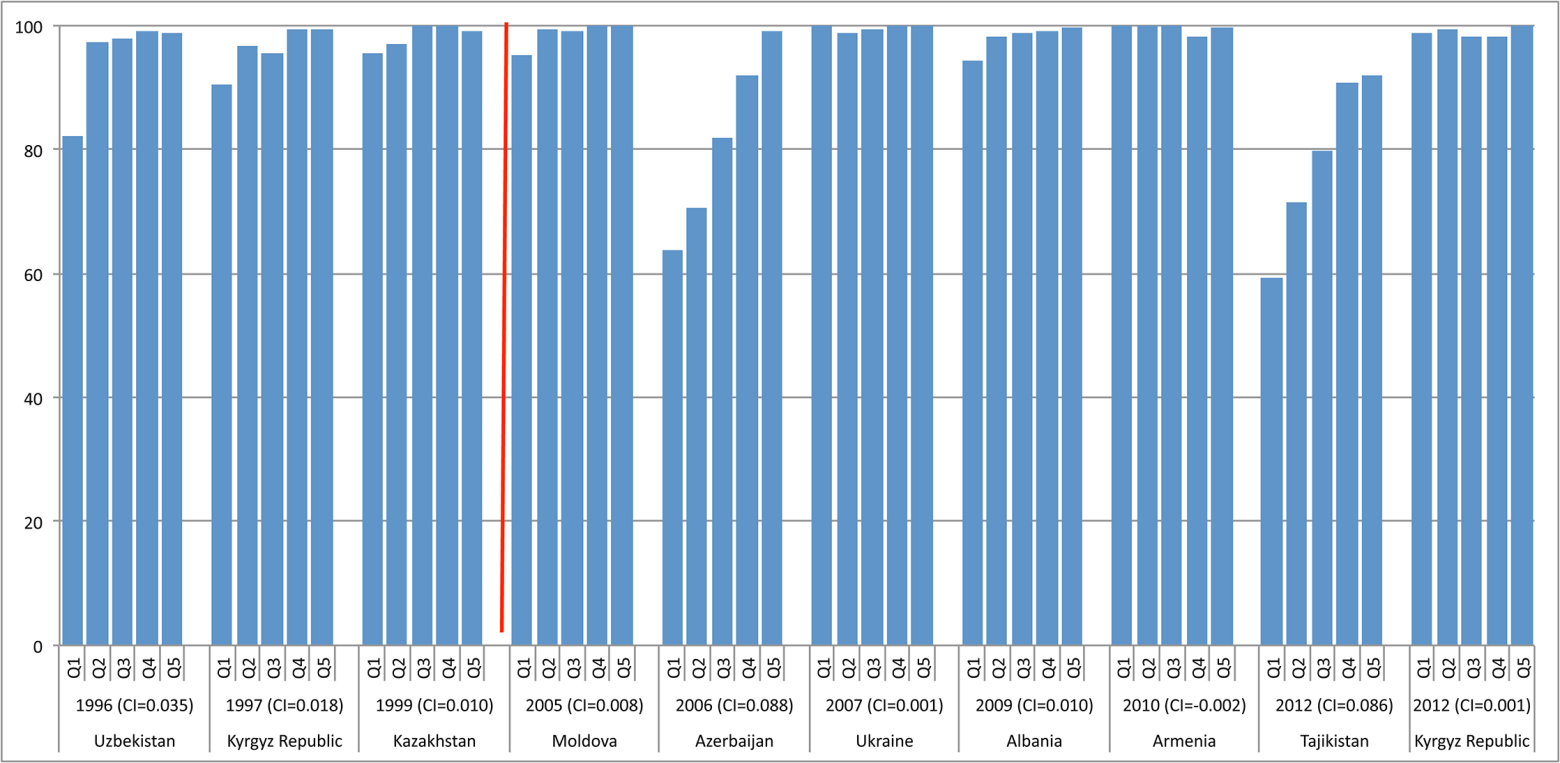
Distribution of facility delivery coverage in select countries in Europe and Central Asia, by wealth quintile, 1996–2012. Q1-5 = wealth quintiles from poorest to wealthiest households. CI = concentration index.

Fig 3 shows that in Europe and Central Asia, equity has been relatively high since the early 1990s. For example, prior to the millennium, the three countries from Central Asia that had surveys during that time period already had relatively low inequities for facility delivery, i.e., CI<0.10, (Kazakhstan, Kyrgyz Republic, and Uzbekistan). Although DHS have been sporadic in that region, the countries with surveys in the last decade (Albania, Armenia, Azerbaijan, Kyrgyz Republic, Moldova, Tajikistan, and Ukraine) all registered low levels of inequality for facility delivery. The same is true for family planning indicators. Turkey, with only one DHS survey during the last 25 years, has also been relatively equitable (CI<0.17) for all indicators.

There are several factors likely responsible for relatively high equity in this region. First, in former Soviet countries, education levels are relatively equitable, given the high level of investment in education under the Soviet regime. Additionally, most of these countries have a long history of free health care, with relatively strong health care systems in place. Overall use of MCH services is also high, with almost all women using facility delivery in most countries, thereby explaining high equity in this region. A final explanation for higher equity in Central Asia is that fertility rates are much lower than in other parts of the world, making it easier to ensure equity of reproductive and maternal health services. We do see more notable inequities in Azerbaijan (2006) and Tajikistan (2012), the reasons for which were not explored in this study.

#### Middle East and North Africa

**Fig 4:**
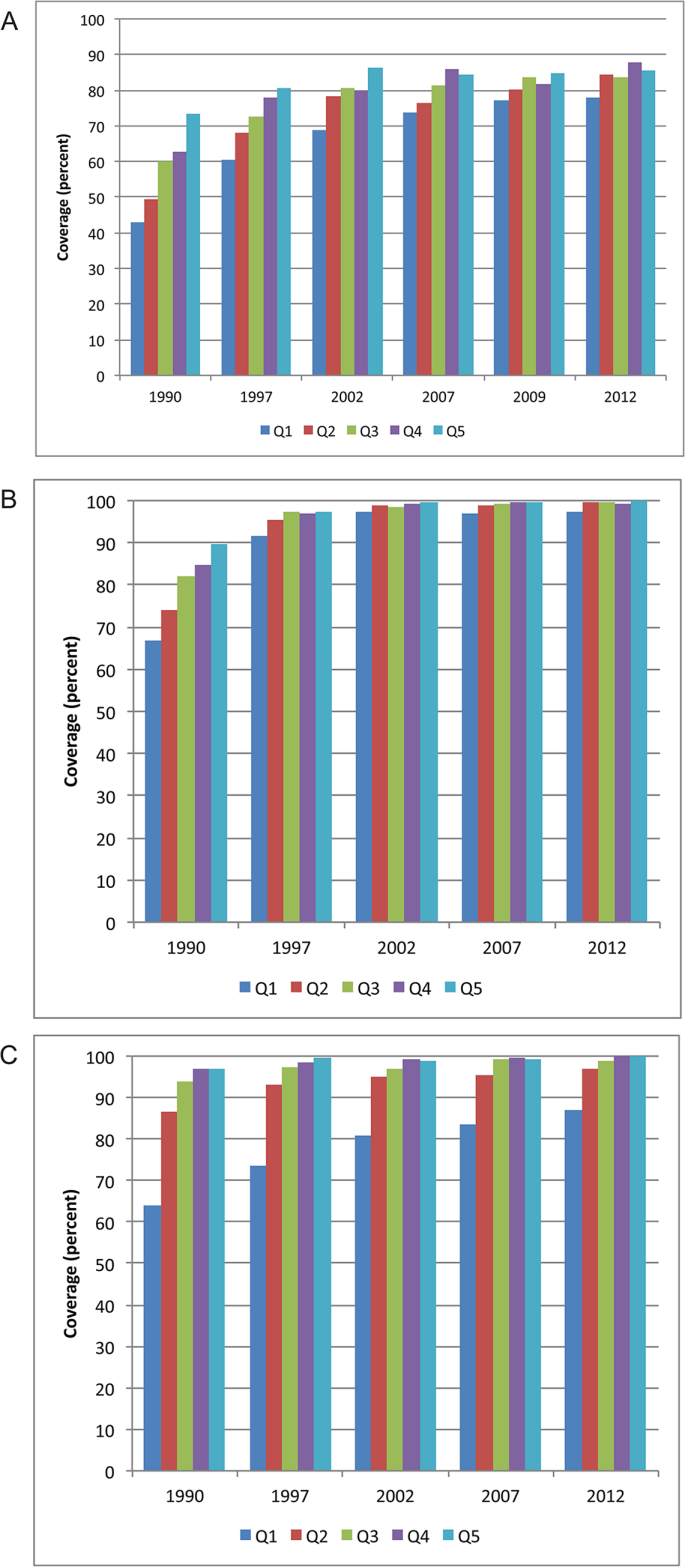
Distribution of reproductive and maternal health service coverage in Jordan, by wealth quintile, 1990–2012. (A) Demand met for family planning. (B) Antenatal care. (C) Facility delivery. Q1-5 = wealth quintiles from poorest to wealthiest households. CI = concentration index.

**Fig 5:**
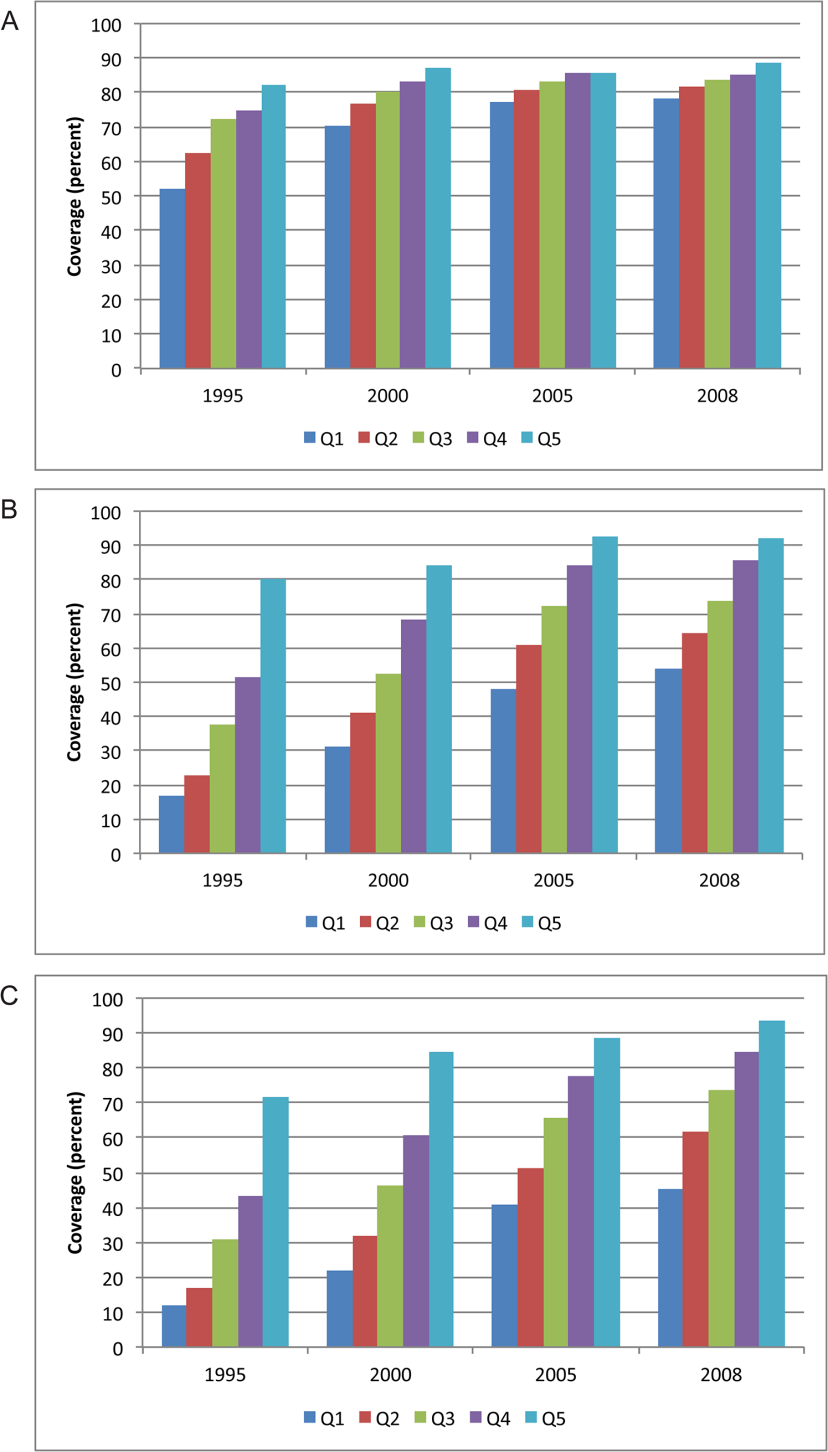
Distribution of reproductive and maternal health service coverage in Egypt, by wealth quintile, 1995–2008. (A) Demand met for family planning. (B) Antenatal care. (C) Facility delivery. Q1-5 = wealth quintiles from poorest to wealthiest households. CI = concentration index.

Although there have been fewer surveys in the Middle East and North Africa than in other regions, the findings are also mixed, with Jordan leading the way in terms of equity across all indicators. Since the early 1990s, Jordan has had high coverage of family planning, ANC and facility delivery overall, but over time the country has made tremendous progress in increasing coverage among the poorest population, as shown in Fig 4. Egypt, on the other hand, has experienced high equity of its family planning indicators but there are still relatively large disparities in use of ANC and facility delivery: just over half of the poorest quintile was using ANC and facility delivery, while nearly 100% of the wealthiest quintile used these services (Fig 5). Morocco’s two surveys also demonstrate substantial improvements in equity between 1992 and 2003–04.

These findings (Figs 4 and 5) show how neighboring countries, with similar cultures and economies can have markedly different access to health services. Jordan, which contributes much more of its government budget to health, is also one of six countries in the Middle East that has specifically adopted health-equity oriented policies and made clear statements about equity in their health strategic plans [40]. The fact that Jordan is performing so well in terms of equity of maternal health suggests the importance of the Ministry of Health’s strong stewardship role in bringing about this change during the last 25 years.

#### Latin America and Caribbean

**Fig 6:**
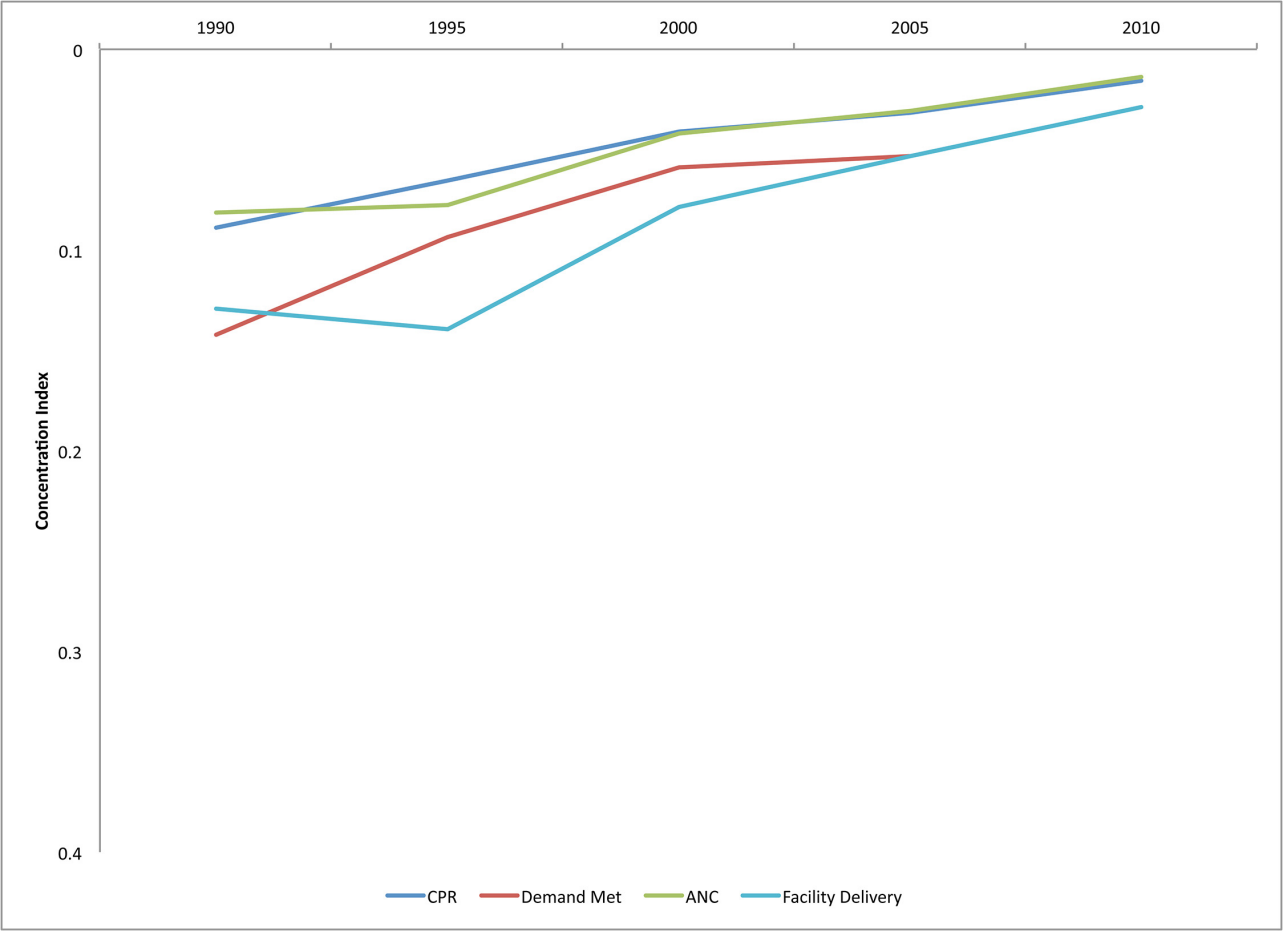
Equity of reproductive and maternal health service coverage in Colombia, 1990 to 2010. CPR = contraceptive prevalence rate. ANC = antenatal care.

Although DHS data are also limited in Latin America and Caribbean, the findings from the region suggest that tremendous progress has been made at improving equity of RH and MH service coverage, with Brazil and the Dominican Republic recording some of the most equitable results for all indicators, even though the latest DHS from Brazil was in 1996. Colombia has maintained high equity across all indicators since the early 1990s and in the most recent time period registered almost perfect equity across all indicators (Fig 6).

While there are too few countries in our sample to draw general conclusions, much of the progress in the countries studies is likely linked to the strong health systems that have been put in place, and the relatively high extent of risk-pooling. Many Latin American countries have pursued the goal of UHC for decades, and their relatively strong health systems help to increase access to key priority services such as family planning, ANC and facility-based care. For example, Brazil’s move to UHC has taken a “bottom-up” approach to health care reform and for decades has had strong public health programs in place at the local level. The country’s approach to targeting the poorest populations embodies the principals laid out in the 1988 constitution that establishes universal and egalitarian access to health care as a fundamental right of citizens and an obligation of the state [41]. Colombia’s history of equitable RH and MH utilization is likely linked to the country’s high coverage of health insurance. Despite progress made in Colombia, however, there are features of the health system that create challenges for completely closing the equity gap in MH, including lack of focus on primary care or health promotion, as well as laws and policies that restrict provision of labor and obstetrics by midwives and staff trained in MH [42].

Although the Dominican Republic and Haiti share the same island, their health systems are markedly different. Haiti has been highly equitable (CI<0.02) for CPR, unmet need and ANC, but this is largely due to low coverage of services across all quintiles. Use of facility delivery in Haiti, however, was the third least equitable in the sample (CI = 0.33) in the most recent time period. Among the poorest quintile in Haiti, only 10% of women deliver in a facility, compared with 80% of women in the highest quintile. Given the lack of DHS surveys in the region, it was difficult to draw any meaningful conclusions about the Caribbean region’s progress toward more equitable MH service coverage.

#### South and East Asia

**Fig 7:**
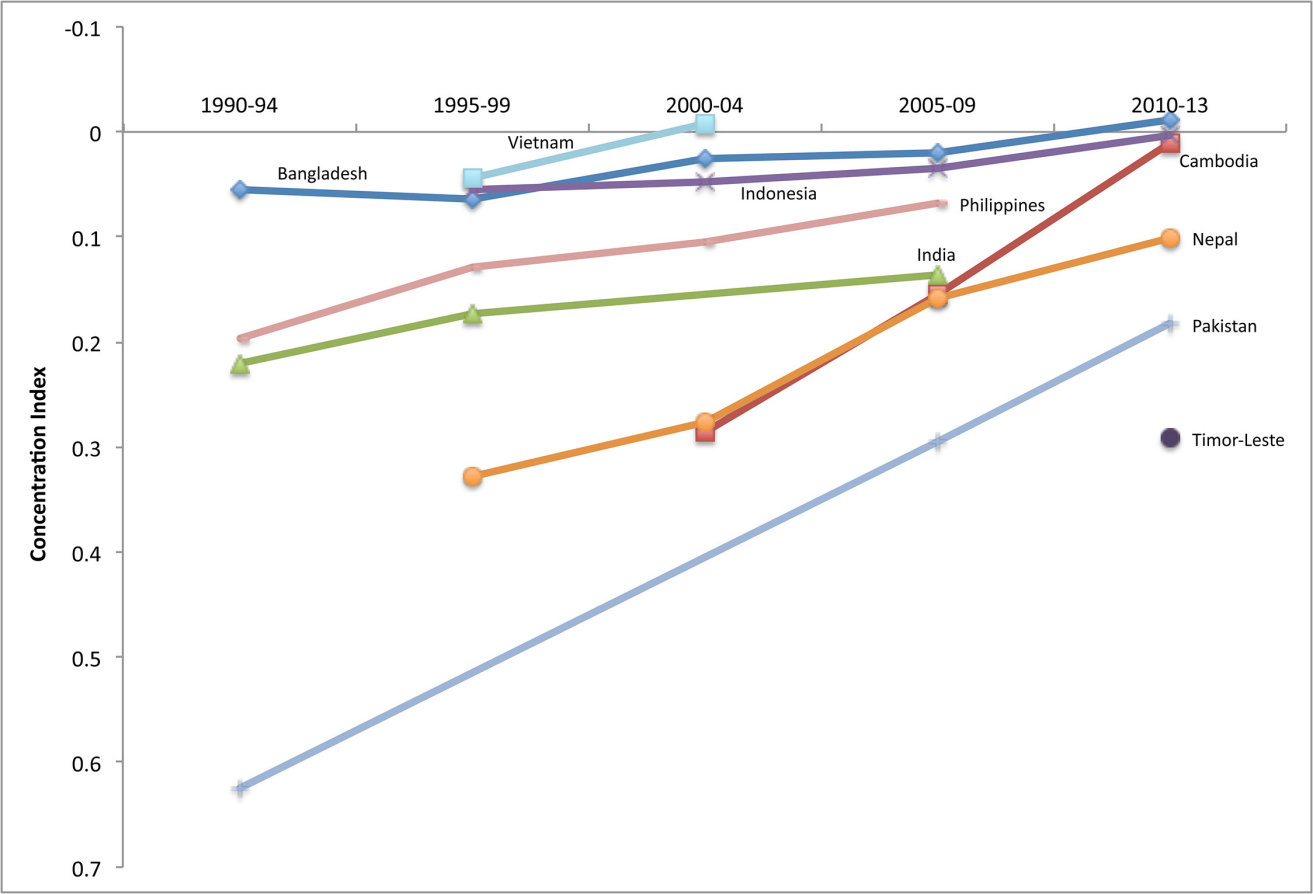
Equity of demand met for family planning in select countries in South and East Asia, 1990–2013.

**Fig 8:**
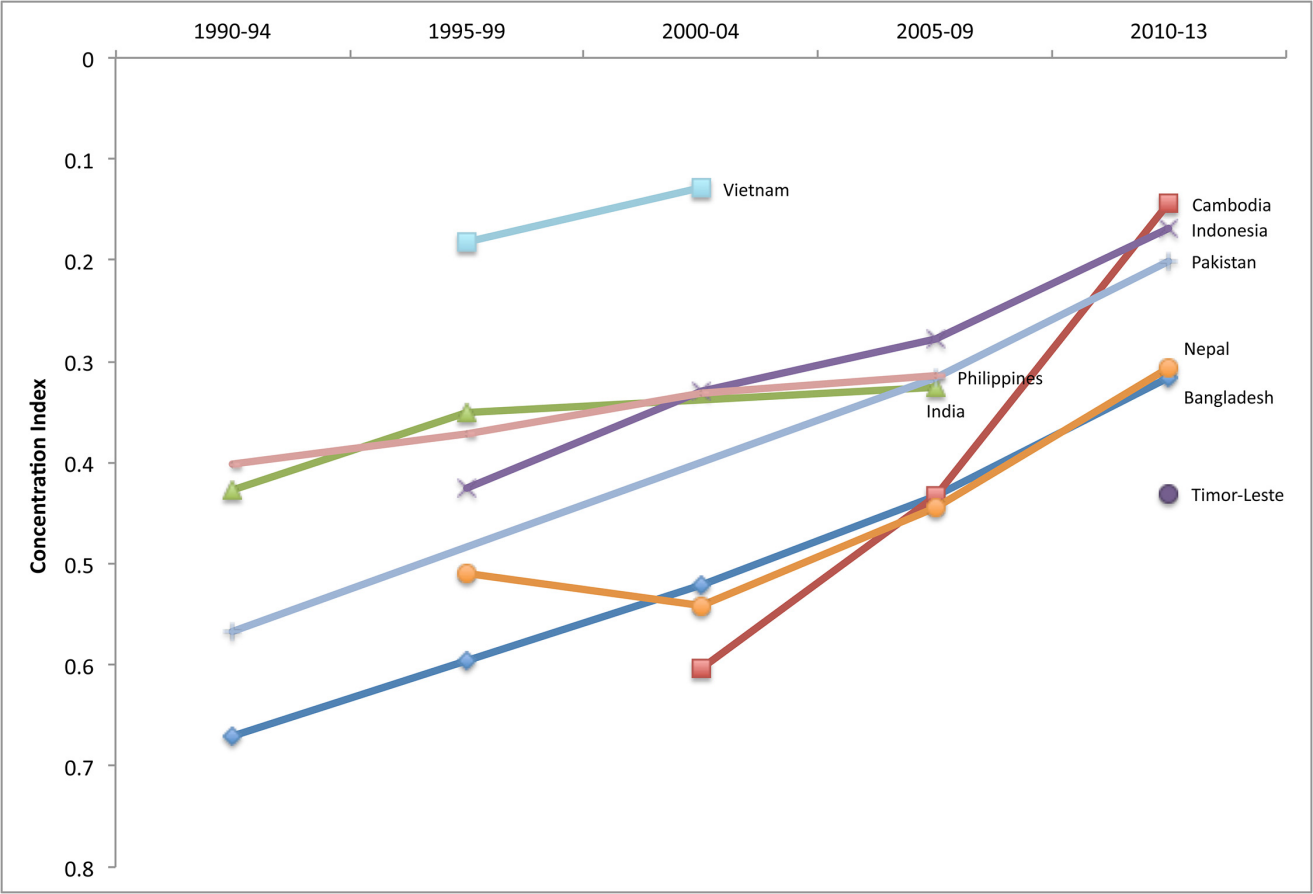
Equity of facility delivery coverage in select countries in South and East Asia, 1990–2013.

Use of DHS in both South Asia and East Asia and Pacific has been somewhat sporadic and has not covered all countries, but the results from the nine surveys available show that before the millennium, use of facility delivery was highly inequitable (CI> = 0.40) in every country except Vietnam, where inequities were less stark (CI = 0.18 in 1997). In the early 1990s, Bangladesh was also the least equitable, with Nepal and Pakistan following closely behind. Although not all countries had DHS for all time periods, recent data show that Timor-Leste was one of the least equitable countries in the sample in 2010–12. Figs 7 and 8 depict the equity of family planning and facility delivery coverage in the region.

**Fig 9:**
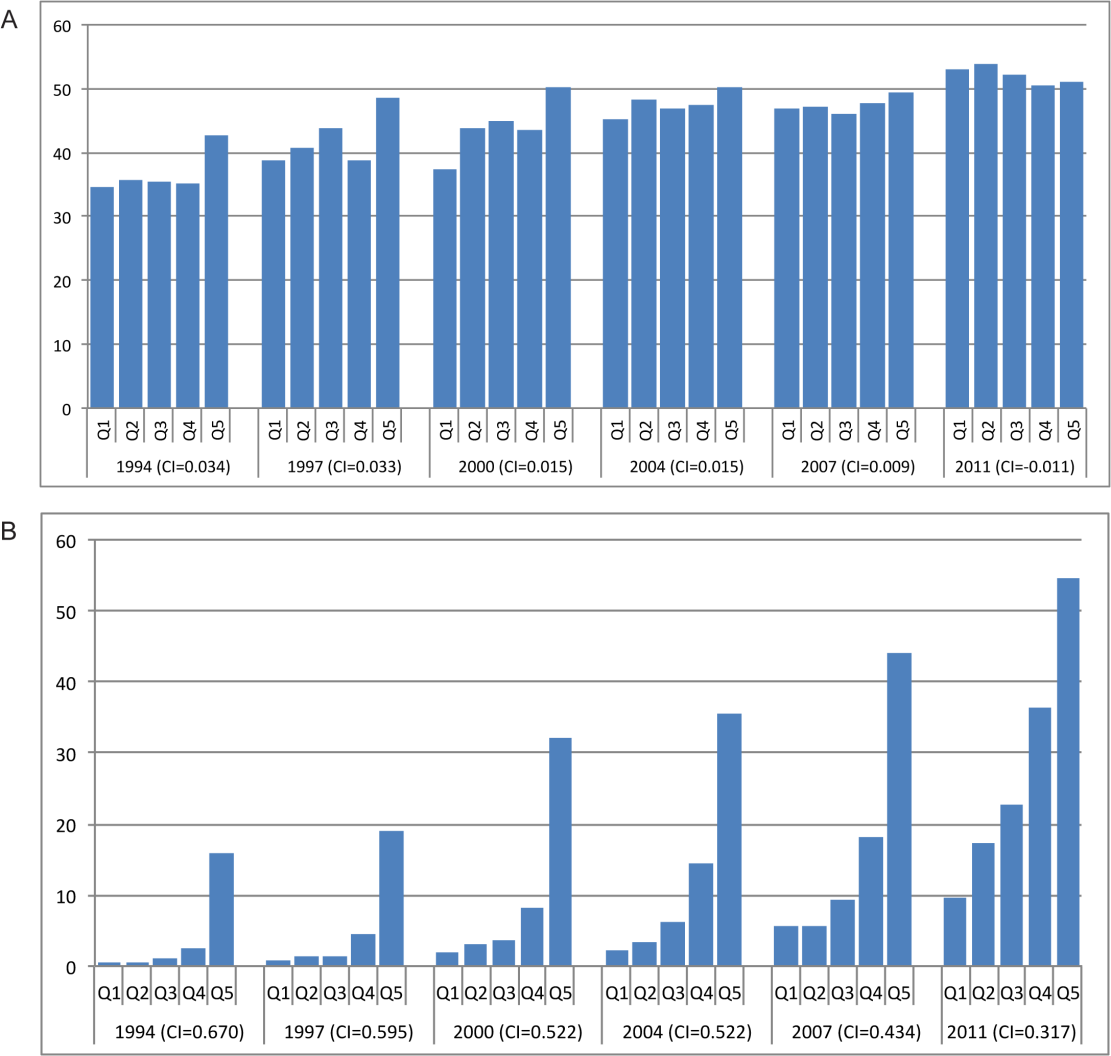
Distribution of reproductive and maternal health service coverage in Bangladesh, by wealth quintile, 1994–2011. (A) Contraceptive prevalence rate. (B) Facility delivery. Q1-5 = wealth quintiles from poorest to wealthiest households. CI = concentration index.

**Fig 10:**
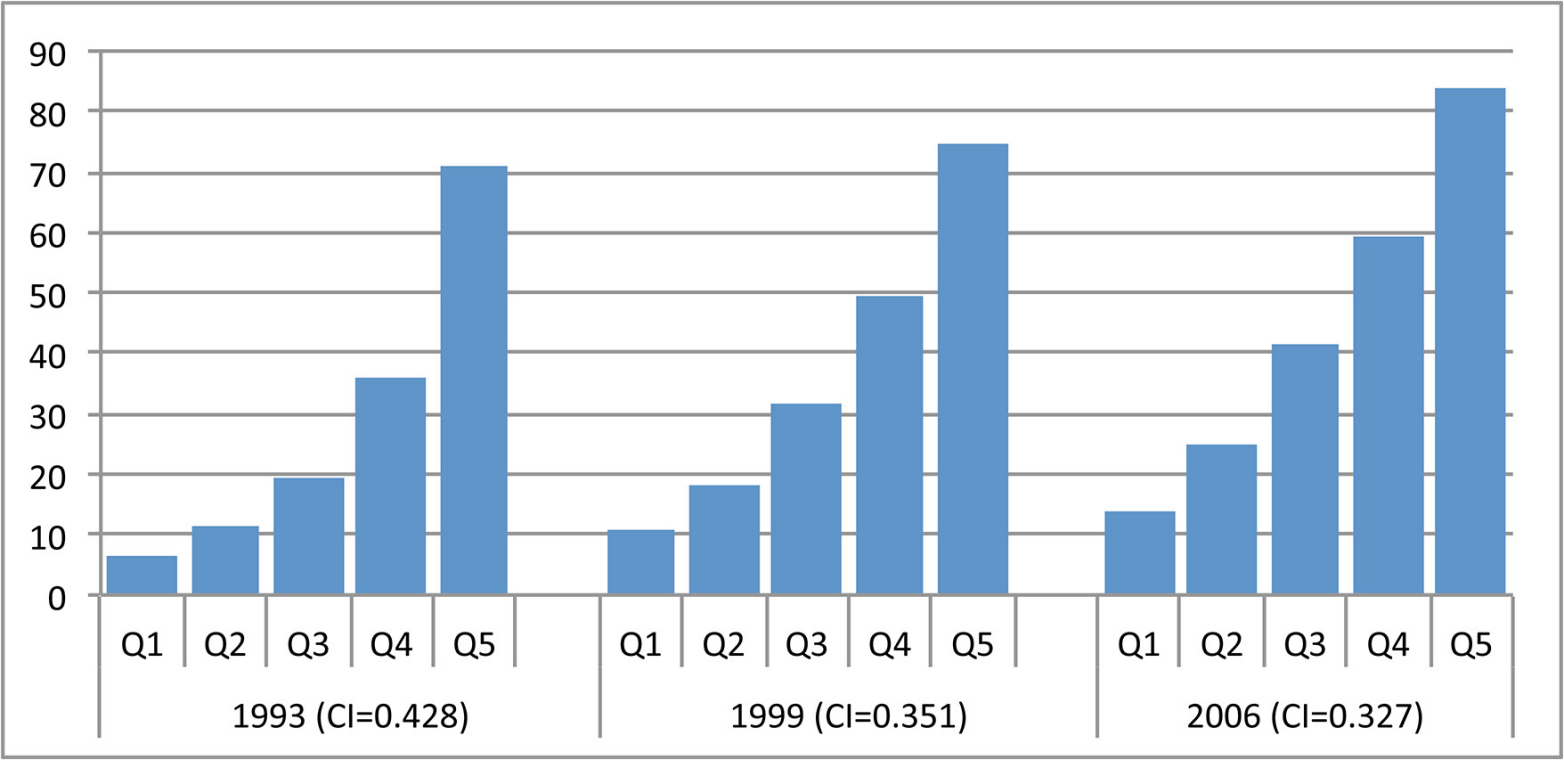
Distribution of facility delivery coverage in India, by wealth quintile, 1993–2006. Q1-5 = wealth quintiles from poorest to wealthiest households. CI = concentration index.

**Fig 11:**
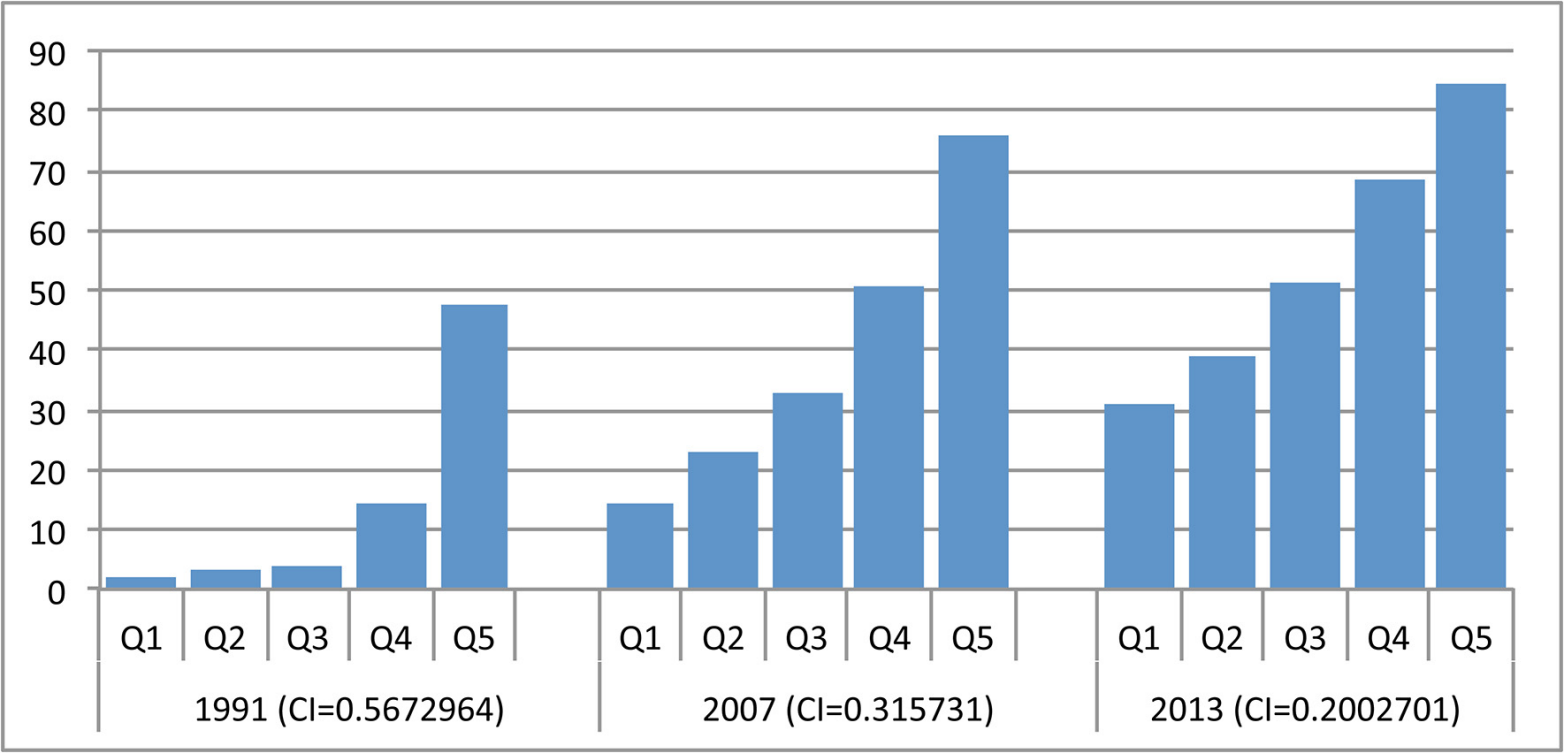
Distribution of facility delivery coverage in Pakistan, by wealth quintile, 1991–2013.

By the last time period, the equity gap in South and East Asian countries had narrowed, particularly for demand met for family planning (Fig 7). Despite progress, inequities in use of facility delivery are still widespread in the region, especially in Timor-Leste, Bangladesh and Nepal (Fig 8). The most noteworthy inconsistency across indicators is Bangladesh, which registered the highest equity for CPR and family planning demand met, but the lowest for facility delivery, despite improvements made on this indicator. Indicators for CPR and facility delivery by wealth quintiles in Bangladesh are shown in Fig 9. India has not had a survey in recent years, but in 2005–06 inequities were still relatively high, particularly for facility delivery. In fact, Fig 10 shows that use of facility delivery in India is still relatively inequitable across socioeconomic groups (CI = 0.33 in 2005–06). Similarly, inequities in the Philippines are still high for facility delivery. Meanwhile, of the countries studied, Pakistan experienced the largest improvement for three of the four services (CPR, demand met, and ANC) (Fig 11 **).** However, much of this improvement in absolute equity is due to the fact that use of facility delivery among the poor was almost non-existent in 1991.

#### Africa

**Fig 12:**
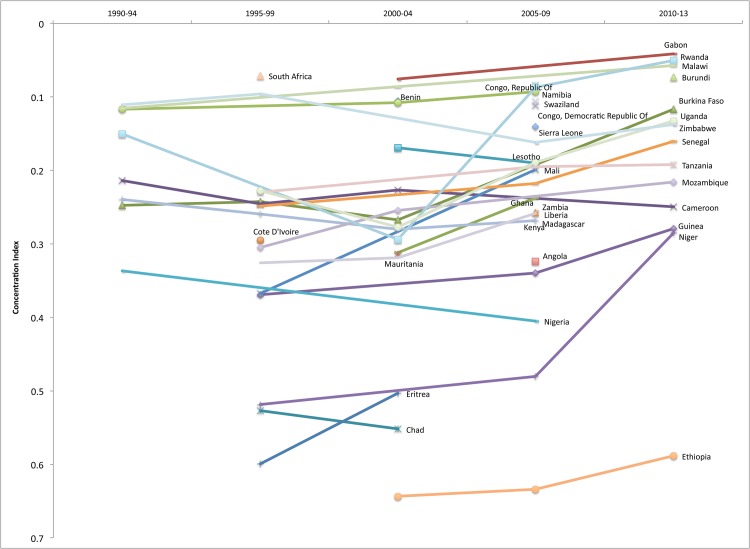
Equity of facility delivery coverage in Africa, 1990–2013.

Africa is the region with the largest number of observations and, given the high TFR and strong USAID-backed RH and MCH programs, has more DHS surveys than other regions. Of the 22 countries with surveys in the 1990s in Africa, five had relatively low inequality levels (CI< = 0.15). These countries are: Malawi, Namibia, Rwanda, South Africa, and Zimbabwe. By the most recent time period, all of these countries had made improvements in equity, with the exception of Zimbabwe, where the inequality of facility delivery worsened between 1994 and 2011. In contrast, Chad, Eritrea, Ethiopia, and Niger were among the least equitable in the sample for facility delivery (CI>0.50) before the millennium, though Niger made considerable progress by its most recent survey. Fig 12 depicts Africa’s equity of facility delivery over time.

While it is not possible to try to explain all the findings from Africa, the results in Rwanda are worth discussing in more detail given the major progress that has been made in reducing inequities of maternal and other health services. Rwanda’s progress likely has much to do with many factors. First, Rwanda is among the African countries with the highest insurance coverage, which was more than 90% in 2010 [43], and is one of the best success stories of a system that has increased coverage through CBHI, which is now mandatory. Additionally, government stewardship, and the strong linkage between health insurance coverage and poverty reduction were likely important factors leading to higher equity of health services more generally. The focused policy framework in Rwanda has allowed donor funding to be channelled to the *mutuelle* program and has strengthened the entire health system [44, 45]. In Francophone Africa the term *“Mutuelle de Santé*,*”* or simply *“mutuelle*,*”* is the equivalent of *Mutual Health Organization*—a synonym for CBHI. Second, the government has taken specific action to improve equity and, through strong financial backing from donors and microfinance institutions, as well as through cross-subsidizations with the formal schemes, has been able to provide sufficient subsidies to stimulate uptake of insurance by the poor and allow gradual expansion of the benefit package [46]. With this support, *mutuelles* have been able to focus on their goals of achieving equity and high coverage levels without the pressure of being self-sustainable. More specifically however, Rwanda’s health care reforms placed considerable attention on maternal and child health and included most services in the benefit package from the very beginning. Despite this progress, however, global experience shows that CBHI has not achieved high coverage rates—largely due to its voluntary nature [47].

### Multivariate analysis

Although the differences in countries with respect to equity of maternal health care are likely due to a complex range of factors, such as a country’s historical political, social and economic structure, we also recognized that factors at the macro level may explain higher equity of RH and MH service coverage. S1 Table shows regression results of the association between the various potential factors and the equity of coverage for the four health services. The results are quite consistent across all four indicators.

Across the four services, education and political commitment are the two most prominent factors that are significantly associated with the four concentration indices, confirming the importance of education in improving maternal health. There is considerable evidence that ensuring universal education of a population can have spillover effects in that it leads to a healthier, more informed population but also improved equity of health [48]. The results also indicate that the association between use of family planning and education is stronger than the association between use of facility delivery or antenatal care.

Government spending on health as a share of total government spending was used as a proxy for political commitment to health and was significantly associated with higher equity of the FP indicators, as well as facility delivery. The association was strongest for demand met.

Results relating the level of prepayment and equity of service coverage were mixed. Compared to out-of-pocket spending, the expansion of prepaid schemes, whether in general or private, coincided with improved equity, but only the associations for ANC were statistically significant. This may be because the level of prepayment is a poor proxy for the extent to which the poor benefit from risk pooling. In other words, increasing prepayment alone is a necessary but insufficient step toward improving the equity of service coverage. How inclusive risk pools are across income levels, as well as the degree of fragmentation among prepaid schemes, are important complements to the level of prepayment in contributing to equity. The secondary data we utilized does not account for these factors, limiting the explanatory power of our model. Finally, prepayment may be more relevant to coverage equity for some services than others. For instance, family planning services and commodities are not always covered by prepayment schemes, and even when they are, people may choose to pay for them out of pocket in private clinical and retail settings, in which case increasing prepayment would not be expected to affect equity in CPR and demand met.

Notably, income (measured as GDP per capita) was not significantly associated with equity, suggesting that economic development is not sufficient for improving health for all, and that targeted approaches to improving health of the poor and other disadvantaged groups should be taken into consideration in countries’ strategies toward sustainable development. In fact, the best examples of improvements in health equity are not from countries in which economic growth has been highest, but instead from countries where targeted reforms have been put in place to cover the poor, e.g., Cambodia.

We did not find that governance was associated with equity of maternal services coverage. Studies have showed that governance is an important factor in translating monetary resources into effective service coverage [21, 26, 27, 30]. However, strong governance does not guarantee that the services delivery is distributed evenly among populations. Countries may start scaling up underutilized services from areas where services are easier to reach, which may result in trade-offs between efficiency and equity. Additionally, the index we used measured governance generally, and not governance in the health sector.

## Conclusions

The results from the analysis confirm what other multi-country studies have found–that equity of MH service coverage varies considerably across countries and over time [4, 48–49]. Our findings show that across the 74 countries studied, equity of reproductive and maternal health service coverage has improved over the last quarter century, but at markedly different rates. In fact, even among the group of countries that are close to achieving the MDGs, progress made on equity varies considerably. For example, Ethiopia has made great strides in reducing MMR, but still has among the highest inequities in use of facility delivery in 2011. Cambodia and Bangladesh have similar MMRs, but have drastically different measures of equity.

A number of country-specific findings are evident from the descriptive analysis. Although each region studied has different social and political histories that no doubt influence access to reproductive and maternal health, it is worth noting that the countries with the highest equity either have systems that have been created on egalitarian principals or have introduced reforms that have had a specific equity focus, e.g., Cambodia’s health equity funds that specifically target the poor within an overall framework of universal health coverage; Rwanda’s CBHI scheme, which was heavily subsidized by the government and donors and allowed the scheme to cover the poor; Latin America’s well-developed health insurance schemes that cover the bulk of the population; Jordan’s schemes, which reflect high levels of government financing and a strong stewardship role with a focus on improving access among the poor; to the majority of countries in Central Asia, where equitable RH and MCH care likely has much to do with the region’s long history of universal access to education and free health care under the Soviet regime.

There are limitations to the multivariate analysis that are worth mentioning. First, GLS analysis of our panel dataset could only include countries for which data for at least two years since 1990 were available. Data availability stems in part from a variety of political and health realities that influence the extent of USAID investment in a country for implementation of DHS, health, and other sectoral programs. Thus, the external validity of the results is compromised. Future analysis would benefit from pooling DHS data with that from other sources, particularly UNICEF’s Multiple Indicators Cluster Surveys. Second, the measurement method of government health financing as a share of total health expenditure lacks reliability across countries; in some countries it may include on-budget financing from donors, whereas in other countries it does not. Third, the determinants of equity of RH and MH services is complex and our model is likely to be missing unobservable factors that influence RH and MH equity, such as social determinants of health, which are known to influence health outcomes [22]. Nevertheless, the analysis is useful in generating suggestive findings that can be used to understand the key macro-level policy indicators that are associated with equity of RH and MH services. Additionally, by focusing on these coverage indicators rather than health outcomes, our analysis does not speak to whether the distribution of good health itself is becoming more equitable. Finally, the nature and quality of services may vary across countries, limiting the interpretability of inter-temporal and cross-country comparisons, as one cannot be sure that merely “using” services will result in more “effective” coverage–a term from the UHC discourse that measures the extent to which service utilization is in line with need and is of sufficient quality to result in improvements in health.

Despite the limitations, our multivariate findings confirm the strong association between education and equitable use of RH and MH care, and was the strongest predictor of coverage of all the independent variables examined. The links between education and RH and MH utilization has been well documented. The findings also show that political commitment as measured by the share of government spending allocated to health, is strongly associated with a more equitable system. It is important to note that this association between higher government spending and higher equity is endogenous; countries with more equitable health financing approaches may spend more of their government funds on health; while more money for health may or may not translate into improved equity, depending on the country’s policies. Nevertheless, with domestic resource mobilization becoming such an important priority in the movement towards UHC, this strong association between government financing and equity is promising. As bilateral and multilateral endeavors—such as the new Global Financing Facility for Reproductive, Maternal, Neonatal, Child and Adolescent Health (RMNCAH) [50]—focus on domestic resource mobilization, governments will be better positioned to achieve coverage among the poorest, but will need to ensure that country’s pro-poor approach to delivering health services is clearly articulated through health financing roadmaps and strategic plans that guide countries toward universal health coverage. However, the evidence shows that increases in financing alone will not trickle down to reach the most vulnerable without a focus on equity. In other words, a strategy based on principles of progressive universalism is needed to ensure coverage increases for the poor as countries move toward UHC [8]. In the post-MDG agenda, it will be important to hold governments accountable for achieving more equitable provision of services, and ultimately more equitable health outcomes, by including health equity indicators in global and national monitoring frameworks. Countries take various approaches to improve equity. It is also informative to understand the heterogeneity of approaches that fit the countries’ political, economic and cultural context.

## Supporting Information

S1 Data Replication data set. (DTA)

S1 Table Factors associated with the inequality of coverage for four health services. ^1^ Measured as the share of government spending on health of the total government budget; † Denominator is total health spending; reference group is out of pocket spending. *p<0.10; ** p<0.05. *** p<0.01.
(PDF)

S2 Table Country surveys included in the study, by World Bank region. (PDF)

S3 Table Variables for multivariate analysis. (PDF)
