# Supplementary material for: Did Equity of Reproductive and Maternal Health Service Coverage Increase during the MDG Era? An Analysis of Trends and Determinants across 74 Low- and Middle-Income Countries
Source: PLoS One. 2015 Sep 2;10(9):e0134905. doi: 10.1371/journal.pone.0134905 (PMC4558013; doi:10.1371/journal.pone.0134905)
Supplement: S1 Table — 1 Measured as the share of government spending on health of the total government budget; † Denominator is total health spending; reference group is out of pocket spending. *p<0.10; ** p<0.05. *** p<0.01. (PDF) [file pone.0134905.s002.pdf]

Factors associated with the inequality of four health services

Variable	CPR	Demand met	Antenatal visit	Facility delivery
ln (GDP/Capita in USD PPP)	0.006	-0.008	0.019	-0.004
Education (percentage point)	-0.005***	-0.006***	-0.001***	-0.003***
Urbanization (percentage point)	0.002	0.002	-0.001	-0.002
Public health spending share†	-0.064	0.041	-0.135**	-0.048
Private (non-OOP) health spending share†	-0.171	-0.108	-0.277***	-0.150
Political commitment[1]	-0.617**	-0.974**	-0.169	-0.846***
Governance factor	-0.009	0.001	0.009	-0.006
Constant	0.461**	0.694***	0.129	0.596**
