# Supplementary material for: Did Equity of Reproductive and Maternal Health Service Coverage Increase during the MDG Era? An Analysis of Trends and Determinants across 74 Low- and Middle-Income Countries
Source: PLoS One. 2015 Sep 2;10(9):e0134905. doi: 10.1371/journal.pone.0134905 (PMC4558013; doi:10.1371/journal.pone.0134905)
Supplement: S2 Table — (PDF) [file pone.0134905.s003.pdf]

**Sub-Saharan Africa**											
Angola	2006-07*†	Benin	1996<br>2001<br>2006<br>2011-12	Burkina Faso	1993<br>1998-99<br>2003<br>2010	Burundi	2010	Cameroon	1991<br>1998<br>2004<br>2011	Central African Republic	1994-95
Chad	1996-97<br>2004	Comoros	1996	Congo, Democratic Republic of	2007	Congo, Republic of	2005	Cote d'Ivoire	1994<br>1998-99<br>2011-12	Eritrea	1995<br>2002
Ethiopia	2000‡<br>2005‡<br>2011	Gabon	2000<br>2012	Ghana	1993<br>1998<br>2003<br>2008	Guinea	1999<br>2005<br>2012	Kenya	1993<br>1998<br>2003<br>2008-09	Lesotho	2004<br>2009
Liberia	2007	Madagascar	1997<br>2003-04<br>2008-09	Malawi	1992<br>2000<br>2004<br>2010	Mali	1995-96<br>2001<br>2006	Mauritania	2000-01	Mozambique	1997<br>2003<br>2011
Namibia	1992<br>2000<br>2006-07	Niger	1998<br>2006<br>2012	Nigeria	1990<br>2003<br>2008	Rwanda	1992<br>2000<br>2005<br>2007-08†<br>2010	Sao Tome and Principe	2008-09	Senegal	1997<br>2005<br>2008-09*†§<br>2010-11
Sierra Leone	2008	South Africa	1998	Swaziland	2006-07	Tanzania	1996<br>1999<br>2004-05<br>2010	Togo	1998	Uganda	1995<br>2000-01<br>2006<br>2011
Zambia	1996<br>2001-02<br>2007	Zimbabwe	1994<br>1999<br>2005-06<br>2010-11								
**East Asia**											
Cambodia	2000<br>2005<br>2010	Indonesia	1997<br>2002-03<br>2007<br>2012	Philippines	1993<br>1998<br>2003<br>2008	Timor-Leste	2009-10	Vietnam	1997<br>2002		
**South Asia**											
Bangladesh	1993-94	India	1992-93	Maldives	2009	Nepal	1996	Pakistan	2006-07		

	1996-97<br>1999-00<br>2004<br>2007<br>2011		1998-99<br>2005-06				2001<br>2006<br>2011		2012-13		
Tajikistan	2012	Turkey	1998	Turkmenistan	2000	Ukraine	2007	Uzbekistan	1996		
**Latin America and Caribbean**											
Bolivia	1994<br>1998<br>2003<br>2008	Brazil	1996	Colombia	1990<br>1995<br>2000<br>2005<br>2010	Dominican Republic	1996<br>1999<br>2002<br>2007	Guatemala	1995<br>1998-99	Guyana	2009
Haiti	1994-95<br>2000<br>2005-06<br>2012	Honduras	2005-06<br>2011-12	Nicaragua	1998,<br>2001	Paraguay	1990	Peru	1991-92<br>1996<br>2000	2004-06<br>2007-08<br>2009	2010<br>2011<br>2012
**Middle East and North Africa**											
Egypt	1995<br>2000<br>2005<br>2008	Jordan	1990<br>1997<br>2002<br>2007<br>2009‡<br>2012	Morocco	1992<br>2003-04	Yemen	1997				

Notes: Two surveys are Malaria Indicator Surveys: Angola 2006-07 and Senegal 2008-09. All others are Demographic and Health Surveys.
* denotes that survey lacked adequate data for CPR
† denotes that survey lacked adequate data for demand met
‡ denotes that survey lacked adequate data for antenatal care
§ denotes that survey lacked adequate data for institutional delivery
