# Supplementary material for: Did Equity of Reproductive and Maternal Health Service Coverage Increase during the MDG Era? An Analysis of Trends and Determinants across 74 Low- and Middle-Income Countries
Source: PLoS One. 2015 Sep 2;10(9):e0134905. doi: 10.1371/journal.pone.0134905 (PMC4558013; doi:10.1371/journal.pone.0134905)
Supplement: S3 Table — (PDF) [file pone.0134905.s004.pdf]

S3 Table. Variables for multivariate analysis.

Variable	Definition	Source
Contraceptive prevalence rate – modern	Share of women aged 15-49, married or in union, currently using modern methods of contraception	STATcompiler (DHS)
Demand met – modern	Share of women aged 15-49, married or in union, with met demand for modern family planning methods	STATcompiler (DHS)
Antenatal care	Share of live births in the three years preceding the survey where the mother received antenatal care from a doctor or other health professional	STATcompiler (DHS)
Assisted delivery	Share of live births in the three years preceding the survey assisted by a doctor or other health professional	STATcompiler (DHS)
Facility delivery	Share of live births in the three years preceding the survey occurring in a health facility	STATcompiler (DHS)
Income	Natural log of gross domestic product per capita at purchasing power parity (PPP) in constant 2011 international dollars	World Development Indicators (World Bank)
Education	Gross secondary school enrollment as share of secondary school-aged population	World Development Indicators (World Bank)
Urbanization	Share of population living in urban areas, as defined by national statistical offices	World Development Indicators (World Bank)
Government share of health expenditure	General government expenditure on health as a percentage of total expenditure on health	Global Health Expenditure Database (WHO)
Private prepaid share of health expenditure	Out-of-pocket expenditure on health as a percentage of total expenditure on health subtracted from private expenditure on health as a share of total expenditure	Global Health Expenditure Database (WHO)
Social security share of health expenditure	Social security expenditure on health as a percentage of total expenditure on health.	Global Health Expenditure Database (WHO)
Non-social security share of health expenditure	Social security share of health expenditure subtracted from government share of health expenditure	Global Health Expenditure Database (WHO)
Political commitment	General government expenditure on health as a percentage of total general government expenditure	Global Health Expenditure Database (WHO)
Governance	Factor scores derived from the analysis of six governance indicators, which include voice and accountability, rule of law, regulatory quality, control of corruption, political stability, and	Worldwide Governance Indicators (World Bank)

	government effectiveness.	
